# Selection of Orphan Rhs Toxin Expression in Evolved *Salmonella enterica* Serovar Typhimurium

**DOI:** 10.1371/journal.pgen.1004255

**Published:** 2014-03-27

**Authors:** Sanna Koskiniemi, Fernando Garza-Sánchez, Linus Sandegren, Julia S. Webb, Bruce A. Braaten, Stephen J. Poole, Dan I. Andersson, Christopher S. Hayes, David A. Low

**Affiliations:** 1 Department of Molecular, Cellular and Developmental Biology, University of California, Santa Barbara, Santa Barbara, California, United States of America; 2 Department of Medical Biochemistry and Microbiology, Uppsala University, Uppsala, Sweden; 3 Biomolecular Science and Engineering Program, University of California, Santa Barbara, Santa Barbara, California, United States of America; Max Planck Institute for Terrestrial Microbiology, Germany

## Abstract

Clonally derived bacterial populations exhibit significant genotypic and phenotypic diversity that contribute to fitness in rapidly changing environments. Here, we show that serial passage of *Salmonella enterica* serovar Typhimurium LT2 (*St*LT2) in broth, or within a mouse host, results in selection of an evolved population that inhibits the growth of ancestral cells by direct contact. Cells within each evolved population gain the ability to express and deploy a cryptic “orphan” toxin encoded within the rearrangement hotspot (*rhs*) locus. The Rhs orphan toxin is encoded by a gene fragment located downstream of the “main” *rhs* gene in the ancestral strain *St*LT2. The Rhs orphan coding sequence is linked to an immunity gene, which encodes an immunity protein that specifically blocks Rhs orphan toxin activity. Expression of the Rhs orphan immunity protein protects ancestral cells from the evolved lineages, indicating that orphan toxin activity is responsible for the observed growth inhibition. Because the Rhs orphan toxin is encoded by a fragmented reading frame, it lacks translation initiation and protein export signals. We provide evidence that evolved cells undergo recombination between the main *rhs* gene and the *rhs* orphan toxin gene fragment, yielding a fusion that enables expression and delivery of the orphan toxin. In this manner, *rhs* locus rearrangement provides a selective advantage to a subpopulation of cells. These observations suggest that *rhs* genes play important roles in intra-species competition and bacterial evolution.

## Author Summary

*Salmonella* Typhimurium is a bacterium that causes intestinal diseases in a number of animals including humans. In mice, this pathogen invades tissues, causing symptoms similar to typhoid fever. In an effort to understand the evolution of this pathogen, we grew *S.* Typhimurium in either liquid broth or in mice for many generations and examined the resulting “evolved” strains to determine if they were different from the original “parent” culture. We found that many of these evolved strains inhibited the growth of the parent after they were mixed together, and that this growth inhibition requires that the evolved and parental cells are in close contact. Genetic analysis showed that this contact-dependent growth inhibition requires Rhs protein, which has a toxic tip. *Salmonella* is normally resistant to its Rhs toxin because it also produces an immunity protein that blocks toxin activity. However, evolved cells have undergone a DNA rearrangement that allows them to express a different Rhs toxic tip that inhibits growth of the parental cells, which lack immunity to it. This allows the evolved cells to outgrow the original parental cells. Our work indicates that populations of *Salmonella* are dynamic, with individuals battling with each other for dominance.

## Introduction

Bacteria often reside in complex communities such as biofilms in which cells from multiple species touch one another in a three-dimensional network [1]. These environments provide opportunities for cellular interactions, yet the mechanisms underlying contact-dependent competition and cooperation have been largely unexplored until recently. A diverse family of YD-peptide repeat proteins mediates at least two distinct forms of contact-dependent competition in Gram-negative and -positive bacteria [2]. The Rhs (rearrangement hotspot) proteins of Gram-negative enterobacteria [3], [4] are large (∼1,400–1,700 residues) toxic effectors that appear to be exported through the type VI secretion machinery. Related WapA (wall-associated protein A) proteins from Gram-positive bacteria are somewhat larger (∼2,200–3,600 residues) [5] and are likely exported through the general secretory pathway [2]. Rhs and WapA proteins are both characterized by sequence-diverse C-terminal regions (Rhs-CT and WapA-CT) that vary considerably between different strains of the same species. Analysis of several Rhs-CTs and WapA-CTs from *Dickeya dadantii* 3937 and *Bacillus subtilis* subspecies revealed that these domains contain the toxin activities responsible for intercellular growth inhibition. All *rhs* and *wapA* genes are closely linked to small downstream open reading frames that encode RhsI and WapI immunity proteins, respectively. These immunity proteins are also sequence-diverse and only protect against their cognate Rhs-CT (or WapA-CT) toxins. Thus, Rhs and WapA represent related, yet distinct, delivery platforms for polymorphic toxin domains [2]. Because different strains typically express unique *rhs-CT/rhsI* (*wapA-CT/wapI*) alleles, these systems collectively form a complex network of toxin/immunity pairs that are thought to mediate inter-strain competition for environmental resources [2].

The *rhs* loci of Enterobacteriacae often contain one or more additional *rhs-CT/rhsI* gene pairs located downstream of the main *rhs/rhsI* pair. These modules have been termed “orphan” toxin/immunity pairs, because the *rhs-CT* coding sequences resemble displaced fragments from full-length *rhs* genes [6]. Orphan *rhs-CT* genes often contain some coding sequence for portions of the conserved N-terminal regions, but orphan fragments are much smaller than full *rhs* genes and usually lack translation initiation signals. Therefore, it is unclear whether orphan *rhs-CT* genes are expressed, raising the question of whether these auxiliary elements are functional. Here, we show that repeated passage of *Salmonella enterica* serovar Typhimurium LT2 (*St*LT2) produces “evolved” lineages that deploy the orphan Rhs-CT toxin to inhibit the growth of ancestral cells. We provide evidence that the *rhs* locus undergoes rearrangement to fuse the *rhs^main^* and *rhs-CT^orphan^* genes, thereby providing a mechanism to express and export the Rhs-CT^orphan^ toxin domain. These results indicate that *rhs* rearrangement provides a selective advantage to a subpopulation of cells, suggesting that *rhs* plays an important role in clonal selection and bacterial evolution.

## Results

**Figure 1:**
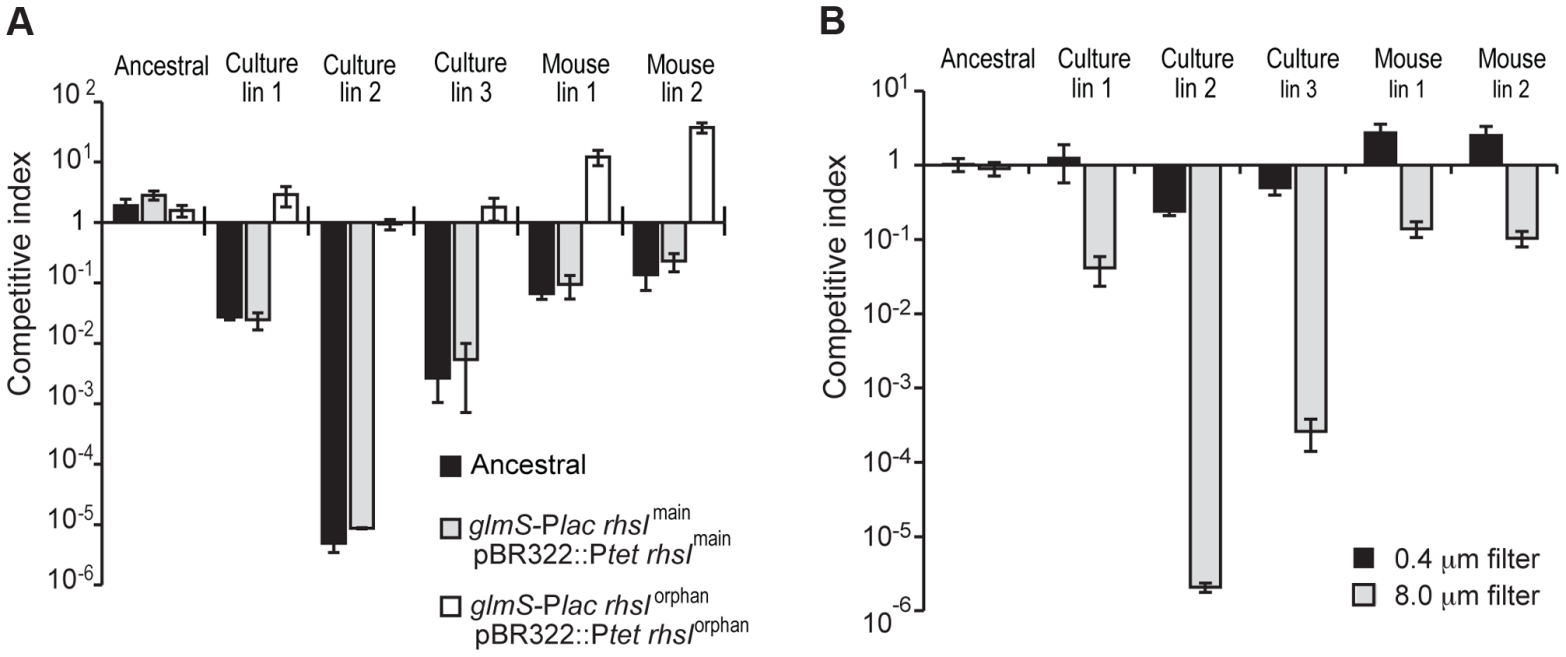
Evolved *St*LT2 cells inhibit the growth of ancestral cells. **A**) The indicated evolved *St*LT2 lineages were co-cultured with the ancestral strain for 24 h in broth. Viable cell counts for each population were determined as colony forming units and these data were used to calculate the competitive index as described in Methods. Each evolved lineage was competed against ancestral cells (black bars), ancestral cells overexpressing *rhsI^main^* (light grey bars) and ancestral cells overexpressing *rhsI^orphan^* (white bars). Reported values represent the mean ± SEM for at least three independent experiments. **B**) The growth inhibition activity of evolved *St*LT2 requires cell-cell contact. Evolved cells were co-cultured with ancestral cells in adjacent wells of a *trans*-well incubation chamber. Culture chambers were separated by membranes containing 0.4 µm or 8 µm pores as indicated. Reported competitive indices represent the mean ± SEM for three independent experiments.

**Figure 2:**
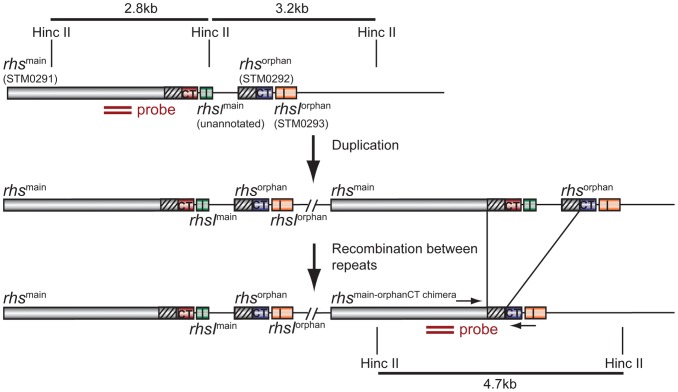
Proposed mechanism of *rhs* locus rearrangement in evolved inhibitor cells. The *St*LT2 *rhs* locus contains a full-length *rhs^main^* gene (STM0291) and an *rhs-CT^orphan^* gene fragment (STM0292) together with associated *rhsI* immunity genes. Duplication of the *rhs* locus would provide the opportunity for subsequent homologous recombination between the 522 bp region of near sequence identity (95%) between *rhs^main^* and *rhs-CT^orphan^* (depicted as diagonal hatched regions). The binding site of the Southern blot hybridization probe is indicated by the double ochre bars. Primer binding sites for PCR amplification of the *rhs^main^/rhs-CTorphan* junction are indicated by convergent horizontal arrows.

In an effort to isolate *St*LT2 strains with increased fitness, we serially passaged cells for ∼1,000 generations in LB medium [7]. Analysis of six independently evolved cultures revealed that each lineage outcompeted ancestral *St*LT2 cells in co-culture experiments (Figures 1A & S1A). Remarkably, we observed the same competitive advantage in four of eight *St*LT2 lineages that were obtained by passage through multiple mouse hosts [8] (Figures 1A & S1B). This competitive advantage was not due to faster growth rate, because four of the evolved lineages grew more slowly than the ancestral strain (Figure S2). To further explore this phenotype, we tested whether evolved lineages inhibit ancestral cells in a contact-dependent manner. We co-cultured evolved and ancestral cells using *trans*-well culture dishes, in which the two populations are separated by membranes of different porosities [9]. The growth of ancestral cells was inhibited when the populations were separated by a cell-permeable 8.0 µm filter, but not when cell contact was prevented with a 0.4 µm filter (Figure 1B). These results indicate that evolved cells must be in close proximity to target cells in order to inhibit growth. This phenomenon is reminiscent of Rhs-mediated growth inhibition, which we recently characterized for *D. dadantii* 3937 [2]. *St*LT2 contains a single *rhs* locus, which contains a full-length “main” *rhs* gene (STM0291) and an “orphan” *rhs* gene fragment (STM0292) (Figure 2). Both *rhs* genes are closely linked to small open reading frames representing potential *rhsI* immunity genes (Figure 2), although the predicted *rhsI^main^* immunity gene found downstream of *rhs^main^* is not annotated in the genome sequence NC_003197. To determine if the *rhs* region is responsible for the observed growth inhibition, we tested whether over-expression of either *rhsI^main^* or *rhsI^orphan^* immunity genes provided protection against evolved *St*LT2 lineages. Parental *St*LT2 cells overexpressing *rhsI^main^* were still inhibited by the evolved lineages, but overexpression of the *rhsI^orphan^* gene fully protected targets from growth inhibition (Figure 1A). These data strongly suggest that evolved *St*LT2 cells gained the ability to deliver Rhs-CT^orphan^ toxin into neighboring cells.

**Figure 3:**
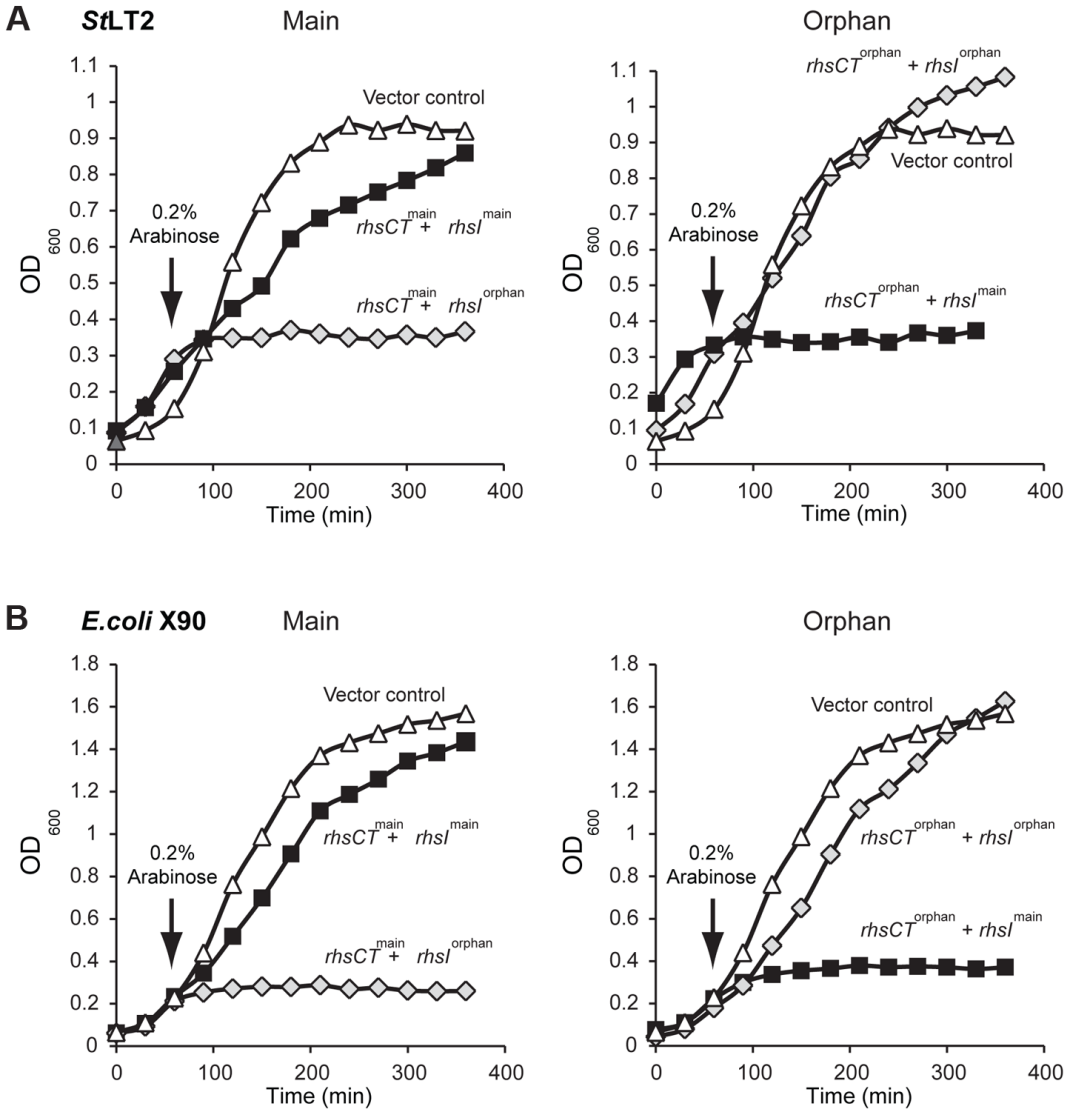
The *St*LT2 *rhs* locus encodes cognate toxin/immunity pairs. **A**) Expression of *rhs-CT^main^* or *rhs-CT^orphan^* inhibits the growth of *St*LT2 cells. Expression the plasmid-borne *rhs-CT* genes was induced by addition of L-arabinose at the indicated time, and cell growth was monitored by measuring the optical density at 600 nm (OD_600_). The cells also co-expressed either *rhsI^main^* (dark squares) or *rhsI^orphan^* immunity genes (light grey diamonds) from IPTG-inducible promoters. Growth is compared to control cells that carry the empty vector plasmids (triangles). **B**) Expression of *rhs-CT^main^* or *rhs-CT^orphan^* inhibits the growth of *E.coli* cells. The *rhs-CT* and *rhsI* genes were expressed in *E. coli* cells from the same plasmids described in panel **A**, and growth monitored by measuring the OD_600_ of the cultures.

We next tested each *rhs/rhsI* gene pair to confirm that they encode functional toxin and immunity proteins. Nucleotides 3608 to 4095 of *rhs^main^* and nucleotides 269 to 741 of *rhs-CT^orphan^* were cloned under the control of the arabinose-inducible P_BAD_ promoter. The predicted *rhsI* immunity genes were cloned using a compatible plasmid under control of the IPTG-inducible P*_trc_* promoter. These plasmids were then introduced into *St*LT2 cells to evaluate toxin and immunity functions. Induction of either *rhs-CT^main^* or *rhs-CT^orphan^* in *St*LT2 resulted in rapid growth arrest (Figure 3A). In each instance, growth inhibition was neutralized by expression of the cognate *rhsI* immunity gene. However, co-expression of non-cognate immunity genes did not alleviate growth arrest (Figure 3A), demonstrating that RhsI^main^ and RhsI^orphan^ immunity proteins are specific for their cognate toxins. We obtained essentially identical results upon expressing the *rhs* main toxin and immunity genes in *E. coli* cells (Figure 3B). These results indicate that Rhs-CT^orphan^ is capable of inhibiting bacterial growth and support a model in which evolved *St*LT2 lineages deploy the orphan toxin to inhibit the ancestral strain.

**Figure 4:**
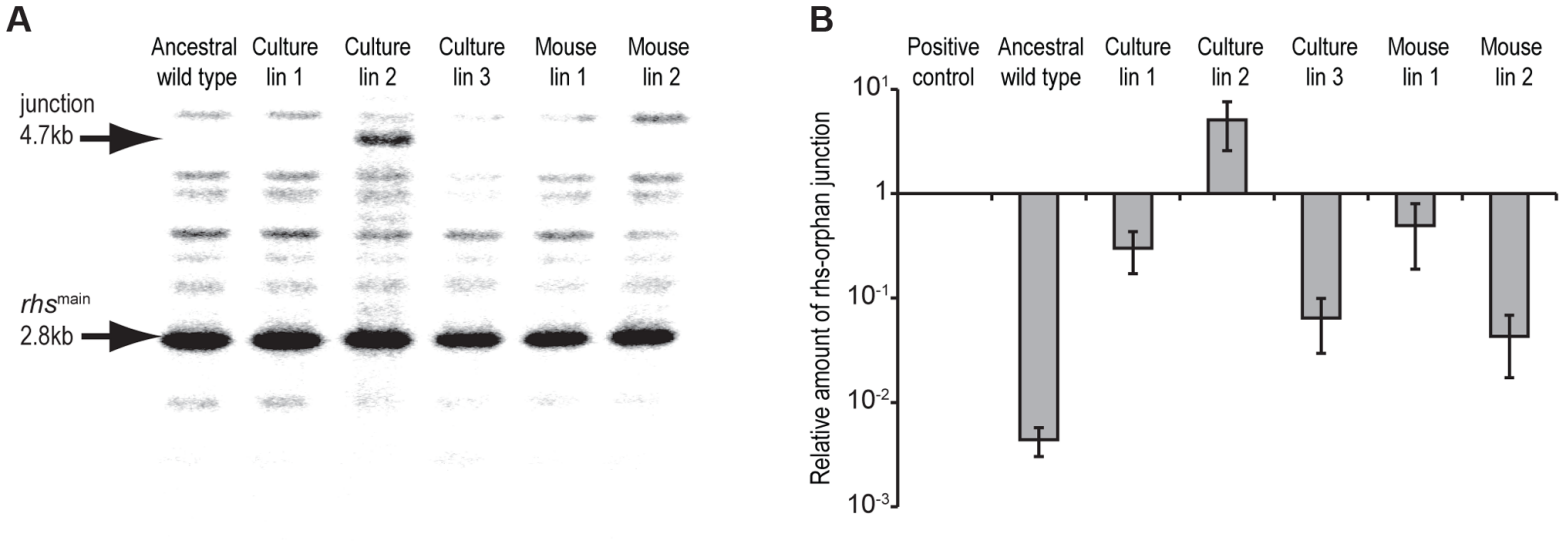
Evidence for *rhs* rearrangement in evolved *St*LT2. **A**) Southern blot analysis of evolved lineages. Arrows indicate positions of the 2.8 kbp HincII restriction fragment containing the ancestral *rhs* region and the 4.7 kbp restriction fragment resulting from recombination between *rhs^main^* and *rhs-CT^orphan^* (see Figure 2 for model). **B**) Real-time qPCR analysis of *rhs^main^/rhs-CT^orphan-CT^* recombination junctions in evolved *St*LT2. The levels of *rhs^main^/rhs-CT^orphan^* junction products are expressed relative to amplified products of a control locus (*bamA*). Positive control cells are engineered *St*LT2 that contain a chromosomal deletion fusing *rhs^main^* to *rhs-CT^orphan^*.

The *rhs-CT^orphan^* sequence does not encode a full-length Rhs protein, raising the question of how this toxin is synthesized and exported from evolved cells. The *rhs^main^* and *rhs-CT^orphan^* coding regions share 95% sequence identity over 522 base-pairs (Figure 2), raising the possibility that homologous recombination in the evolved lines generates a new full-length *rhs* gene that encodes the Rhs-CT^orphan^ toxin domain [10]. Bacteria expressing this Rhs chimera would have a growth advantage if *rhsI^orphan^* expression is low in ancestral cells. However, the proposed recombination event would also delete the *rhsI^main^* gene, rendering the evolved cells sensitive to inhibition by siblings expressing the main Rhs-CT toxin. Therefore, we hypothesized that *rhs* recombination occurs subsequent to duplication of the locus such that evolved cells retain the *rhsI^main^* immunity gene (Figure 2). To test this hypothesis, we analyzed chromosomal DNA from evolved and ancestral lineages by Southern blot. DNA was digested with HincII, which cleaves between the *rhsI^main^* and *rhs-CT^orphan^* coding sequences, and probed with a labeled DNA fragment that specifically hybridizes to *rhs^main^* (Figure 2). We detected a unique junction fragment representing fusion of *rhs-CT^orphan^* to the upstream *rhs^main^* gene in *St*LT2 lineage 2, which displayed the highest level of growth inhibition of all lineages (Figures 1 & 4A). The wild-type *rhs* locus was also detected in lineage 2 (Figure 4A), which is consistent with *rhs* region amplification, but may also indicate distinct populations of recombinant and non-recombinant cells. Orphan *rhs* recombinants were not detected in the other evolved lineages by Southern blot analysis (Figure 4A). Because the growth inhibition phenotype varied in magnitude between the different evolved strains, it is possible that only a fraction of the evolved *St*LT2 cells are *rhs* recombinants. If so, then the proportion of recombined *rhs* loci in the DNA sample may be below the detection limit of Southern analysis. Therefore we analyzed each evolved lineage with quantitative real-time PCR (qPCR) to measure the relative levels of *rhs^main^-rhs^orphan^* junction sequences. All five of the evolved lineages contained 10- to 1,000-fold more *rhs^main^-rhs^orphan^* junction than ancestral *St*LT2 (Figure 4B), consistent with the ability of these strains to deploy Rhs-CT^orphan^ toxin.

**Figure 5:**
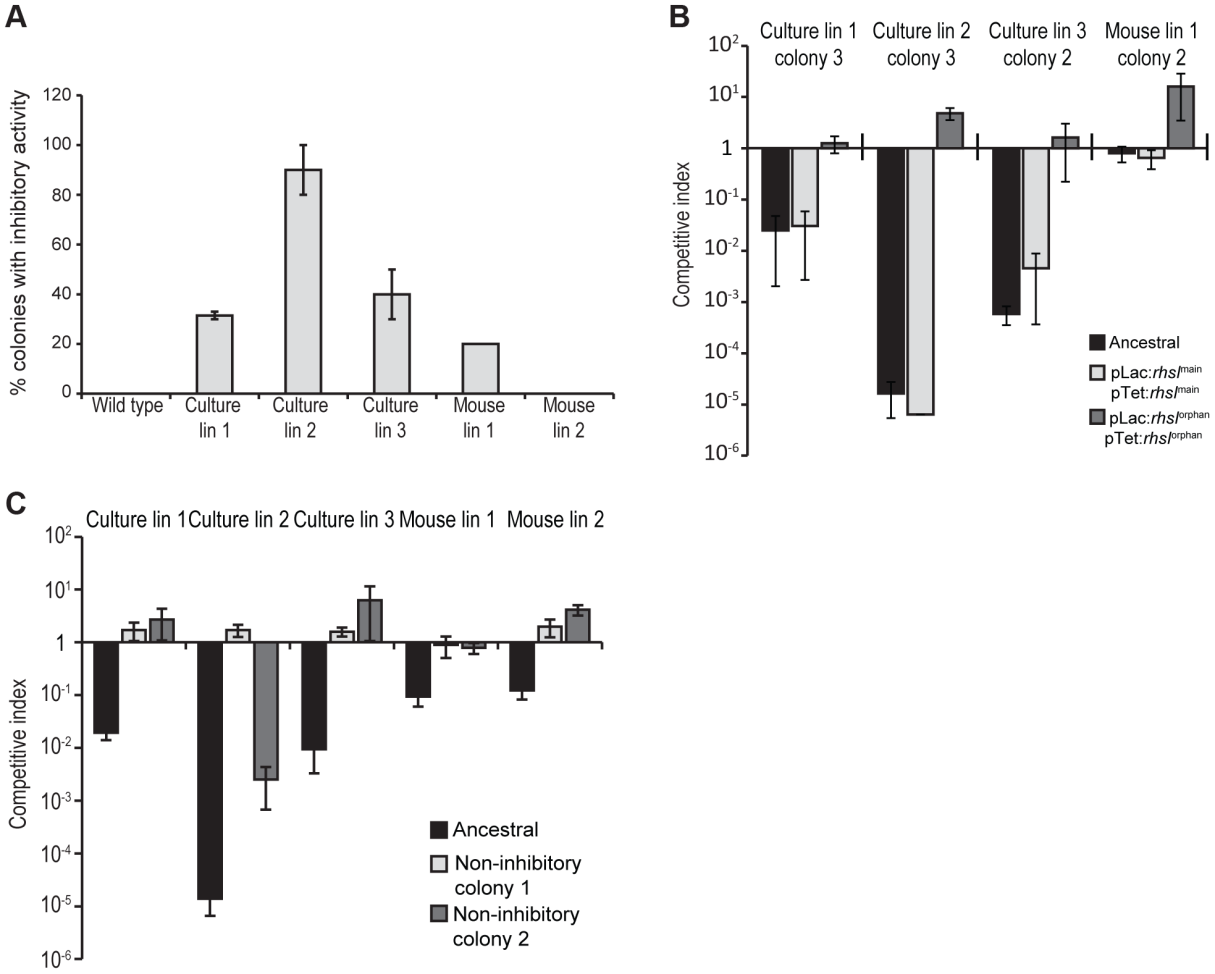
A subpopulation of evolved cells has growth inhibition activity. **A**) Two sets of independent clones were isolated twice from the evolved lineages and tested for growth inhibition activity against ancestral cells. The percentage of evolved clones with inhibition activity is shown. Reported values represent the mean ± SEM for at least two independent experiments. **B**) Growth inhibition activity of isolated evolved clones. Clones from each evolved lineage were competed against ancestral cells (black bars), ancestral cells overexpressing *rhsI^main^* (light grey bars) and ancestral cells overexpressing *rhsI^orphan^* (white bars). Reported values represent the mean ± SEM for at least three independent experiments. **C**) Growth inhibition activity of evolved lineages towards non-inhibitory clones. Evolved lineages were co-cultured with ancestral cells (black bars) and two non-inhibitory clones isolated from the evolved cultures (light grey and white bars). Competitive indices represent the mean ± SEM for at least three independent experiments.

Because only a fraction of the passaged cells appeared to display growth inhibitory activity, we asked whether inhibitor-cell clones could be isolated from each population. As a control, we first isolated colonies from an overnight culture of the ancestral strain and tested these clones for growth inhibition activity. None of the ten ancestral clones tested were inhibitory, suggesting that the proposed *rhs* rearrangements occur at low frequency. By contrast, approximately 30–90% of the clones isolated from the culture-evolved lineages and ∼20% of the clones from mouse-evolved lineage 1 showed inhibition activity against ancestral cells (Figure 5A). However, no inhibitor clones were isolated from mouse-evolved lineage 2 (Figure 5A). Strikingly, the inhibition activity of these clones varied considerably. For example, competitive index values ranged from 10^−1^ to 10^−5^ for competitions between ancestral cells and inhibitory clones isolated from evolved lineage 2 (Figure S3). Although their potencies varied, it appears that each inhibitor-cell clone deployed the Rhs-CT^orphan^ toxin because ancestral cells could be protected through over-expression of *rhsI^orphan^*, but not *rhsI^main^* (Figure 5B). The presence of DNA fragments corresponding to both ancestral and recombinant *rhs* loci in lineage 2 (Figure 4A) suggests that either the *rhs* region was duplicated or there are distinct populations of recombinant and non-recombinant cells. In the latter case, single colonies isolated from the inhibitory lineages would contain only the *rhs*- *rhs*-CT^orphan^ junction and not the *rhs*-CT^main^ sequence. However, PCR analysis of the single colonies with inhibitory activity in Figure 5B showed that each contained both ancestral and recombinant *rhs* loci. In addition, sequence analysis of the recombinant PCR product verified that recombination occurred between the regions of homology shared by *rhs^main^* and *rhs-CT^orphan^*. Together, these data demonstrate that the evolved populations are heterogeneous with respect to Rhs-CT^orphan^ mediated inhibition activity. Furthermore, these results suggest that the inhibition phenotype of a given culture may be due entirely to a minor subpopulation of potent inhibitor cells.

Because inhibitor cells represent a subpopulation in the evolved cultures, the other non-recombinant cells in the cohort are presumably resistant to the Rhs-CT^orphan^ toxin. To test this hypothesis, we isolated non-inhibitory clones from each of the evolved cultures and tested them in competition co-cultures against their respective evolved lineages. As predicted, each of the non-inhibitory clones was either fully- or partially-resistant to its cohort lineage (Figure 5C). These cells likely carry uncharacterized resistance mutations that may prevent cell-cell contact, block the delivery of Rhs-CT^orphan^ toxin,, or increase the immunity of these cells to Rhs-CT toxin.

**Figure 6:**
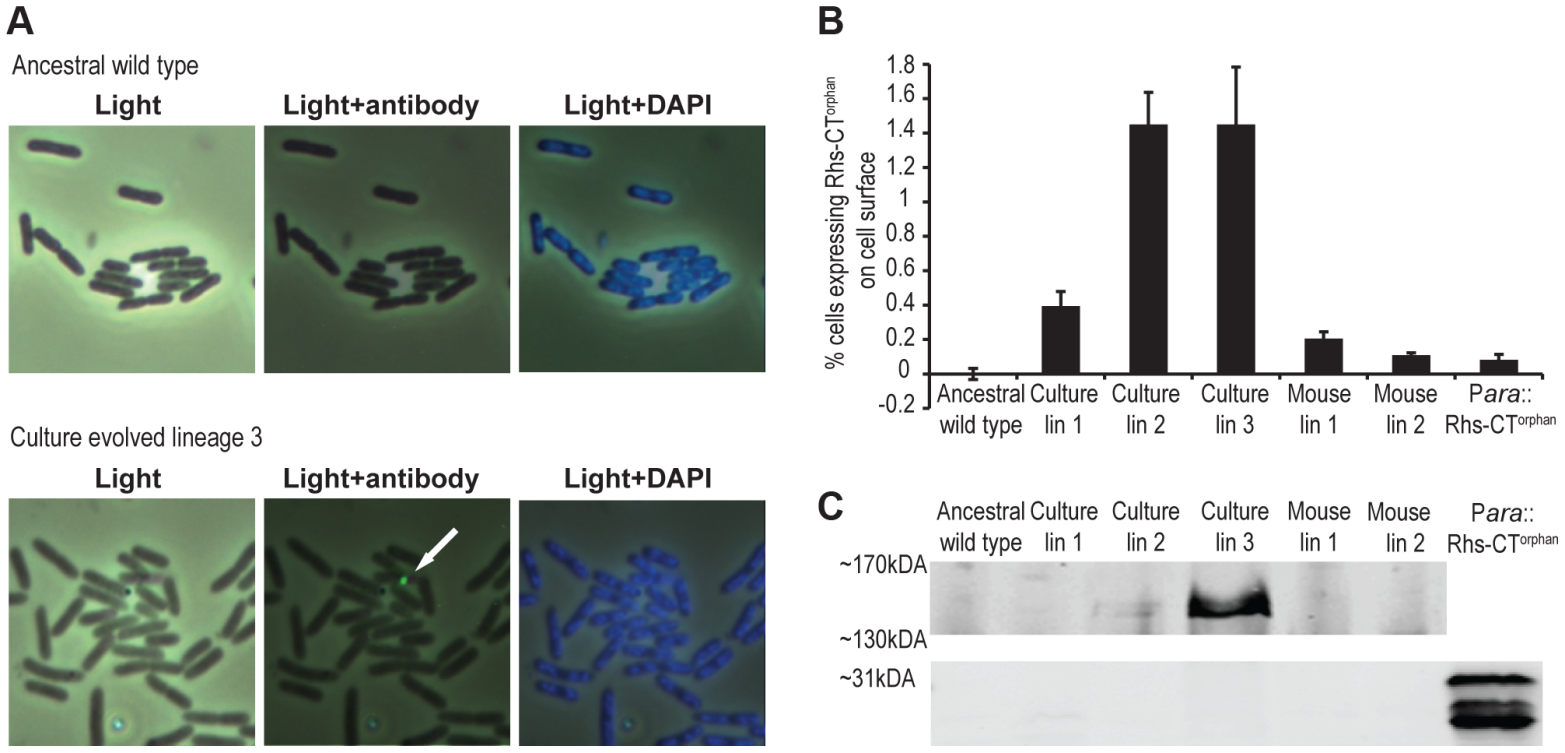
Expression of Rhs-CT orphan in evolved cells. **A**) Immunofluorescence analysis of ancestral *St*LT2 and culture-evolved lineage 3 with antibodies against Rhs-CT^orphan^. Scale is 10 µm×10 µm for each image. **B**) Quantification of surface-expressed Rhs-CT^orphan^. Cells were labeled with Rhs-CT^orphan^ antisera and analyzed by flow cytometry as described in Methods. Reported values represent the mean ± SEM for three independent experiments with 50,000 events recorded per sample. **C**) Immunoblot analysis of Rhs-CT^orphan^. Proteins were isolated from ancestral and evolved cells and analyzed by immunoblot using antibodies against Rhs-CT^orphan^. Regions corresponding to predicted migration positions of Rhs-CT^orphan^ (∼31 kDa) and the chimeric fusion protein (∼151 kDa) are shown.

To directly detect Rhs-CT^orphan^ expression in the evolved lineages, we examined cells by immunofluorescence microscopy using polyclonal antibodies against the Rhs-CT^orphan^ toxin. Rhs-CT^orphan^ antigen was detected on the surface of some cells within evolved lineages 1, 2 and 3 as well as mouse-evolved lineages 1 and 2 (Figures 6A & S4). In contrast, the Rhs-CT^orphan^ signal was undetectable on the surface of both ancestral *St*LT2 cells and cells carrying a deletion of the *rhs-CT^orphan^* (Figures 6A & S4). We then quantified the fraction of cells with Rhs-CT^orphan^ antigen on the cell-surface using flow-cytometry. Evolved lineages showed a 2- to 20-fold increase in the fraction of Rhs-CT^orphan^-positive cells compared to ancestral *St*LT2 cells (Figures 6B & S5). Mouse-evolved *St*LT2 showed very low expression of Rhs-CT^orphan^ antigen on cell surfaces (Figure 6B), consistent with the modest growth inhibition observed for these lineages (Figure 1). Based on Southern blot and RT-qPCR analyses, it seems likely that surface expression of Rhs-CT^orphan^ requires *rhs* locus rearrangement to generate a chimeric *rhs* gene. In accord with this conclusion, we also found that over-expression of *rhs-CT^orphan^* from a multicopy plasmid does not increase Rhs-CT^orphan^ antigen levels on the cell surface (Figures 6B & S5). Therefore, we sought to detect the predicted Rhs fusion protein using antisera to the Rhs-CT^orphan^ toxin. Western blot analysis revealed an immuno-reactive protein at ∼150 kDa in culture evolved lineages 2 and 3 (Figure 6C). This product corresponds to the expected size of the Rhs fusion protein. Moreover, we were unable to detect the 29 kDa product encoded by *rhs-CT^orphan^* in the ancestral and evolved lineages (Figure 6C). Together, these data strongly suggest that the *rhs-CT^orphan^* reading frame must recombine with *rhs^main^* to be expressed.

## Discussion

The results presented here show that serial passage of *St*LT2, in either laboratory media or within a natural host, leads to enrichment of cells that express Rhs-CT^orphan^ toxin. Analysis of the *rhs* locus indicates that evolved cells undergo recombination between *rhs^main^* and *rhs-CT^orphan^*, forming a gene fusion that allows the Rhs-CT^orphan^ toxin domain to be deployed. Rearranged *rhs* genes are detected at low levels within the evolved populations, indicating that only a fraction of cells are recombinant inhibitors. A number of observations argue that this subpopulation of cells is responsible for growth inhibition activity. First, the relative competitive advantage of each evolved lineage is correlated with its level of recombinant *rhs* junctions and surface expression of Rhs-CT^orphan^ antigen. More importantly, ancestral cells are fully protected when they over-express the *rhsI^orphan^* immunity gene. Because Rhs immunity proteins are highly specific for their cognate toxins, this latter result demonstrates that Rhs-CT^orphan^ toxin is indeed deployed by the evolved lineages. This result also indicates that ancestral *St*LT2 cells do not normally express *rhsI^orphan^* immunity genes under laboratory conditions. The number of inhibitor cells within each lineage is not known, but can be estimated to be <2% of the population based on flow cytometry measurements of Rhs-CT^orphan^ antigen on cell surfaces. However, we note that this assay may underestimate the actual number of recombinant inhibitor cells because Rhs effectors are likely exported through type VI secretion systems [2], [11]. Although recent studies indicate that the N-terminal PAAR domain found within many Rhs proteins forms the tip of the type VI injection structure [12], other structural studies show that Rhs-peptide repeats form a chamber capable of encapsulating toxin domains [13]. Therefore, much of the Rhs-CT^orphan^ antigen may be inaccessible to antibody until it is delivered to target cells. In accord with this model, we only detect Rhs-CT^orphan^ where two bacteria make contact with one another and never on the surface of individual cells. Regardless of the absolute number of recombinants or *rhs* expression levels, our results suggest that a small number of inhibitor cells are capable of inhibiting a large excess of ancestral cells. The same phenomenon has been observed during bacterial contact-dependent growth inhibition (CDI), in which each CDI^+^ cell is able to inhibit 100–1,000 target cells over a few hours [9]. Presumably, the unstructured environment in shaking broth culture promotes a series of transient cell-cell interactions, thereby enabling toxin delivery to multiple ancestral cells.

Chromosomal duplications and amplifications occur frequently in bacteria, typically at rates of about 0.1% per generation for any given locus [14], [15]. However, there is a cost to maintaining amplified regions, and gene duplications are lost during segregation at frequencies up to 10% per generation [16], [17]. Therefore, positive selection is required to retain multiple gene copies. If the amplified region can be stabilized, then the additional gene copy can diverge towards a new function, thus providing a mechanism for evolution [16], [18]. Rearrangement of *rhs* loci represents a previously unrecognized mechanism for bacteria to exploit chromosomal amplifications for adaptation. We propose that, subsequent to duplication, homologous recombination occurs between *rhs^main^* and *rhs-CT^orphan^* to generate a novel chimeric *rhs* element. This recombination would necessarily delete one copy of the *rhsI^main^*, but the other copy would remain and ensure that recombinant cells retain immunity to the Rhs^main^ toxin should it be deployed by neighboring non-recombinant siblings. This model also predicts that evolved recombinant cells could undergo homologous recombination to restore the original *rhs* locus (see Figure 2, reverse of the duplication step). Thus, *rhs* rearrangement could be exploited transiently under conditions where it confers a selective advantage, but rapidly revert back to the ancestral genotype as environmental circumstances dictate.

Analysis of over 150 *Salmonella* genomes shows that *rhs-CT* toxin sequences are diverse with at least 57 distinct sequence types (Figure S6A & Table S1). This is a common feature of *rhs* genes in other bacteria as well and suggests that Rhs mediates inter-strain competition. All *Salmonella* serovars contain at least one *rhs* gene, located on pathogenicity islands SPI-6 or SPI-19 [19], [20]. Approximately 50% of these serovars contain at least one predicted *rhs* orphan sequence, with some strains containing as many as eleven modules. There is generally high conservation of Rhs-CT^main^ and Rhs-CT^orphan^. sequences within a given serotype. For example, all sequenced Typhi isolates contain the same Rhs-CT^main^ and Rhs-CT^orphan^. sequences, whereas these CT sequence types are only found in one other serotype, thus suggesting that different toxins are linked to serotype and/or the type of infection. However, orphan *rhs-CT* sequences in one serotype can be present within the main *rhs* gene of another serotype. For example, the *St*LT2 Rhs-CT^orphan^ toxin studied in this work is part of the full-length main Rhs in *Salmonella enterica* serovar Saintpaul SARA23 and some Newport isolates (Figures S6A & S6B). These observations and the association with horizontally transferred elements suggest that *rhs* genes are exchanged between different serovars and contribute to the evolution of toxin diversity.

Given that Rhs toxins are encoded on pathogenicity islands, it seems likely that these systems also play important roles in *Salmonella* growth and fitness during pathogenesis. Indeed, *St*LT2 mutants lacking a chromosomal region containing *rhs-CT^orphan^* are outcompeted by wild-type cells in mice [21], and *St*SL1344 mutants lacking *rhs* are completely attenuated in pig and cattle models of infection [22]. These observations raise the possibility that *rhs* locus rearrangement occurs commonly during infections. Intriguingly, *St*LT2 produces distinct intracellular infection foci, each originating from one or only a few clones [23]. Similarly, analysis of mice orally infected with *Yersinia pseudotuberculosis* indicates that only a few bacterial clones are able to disseminate from the intestines to the spleen and liver [24]. Clonal invasion has also been reported for *Yersinia enterocolitica* infections [25], but the mechanisms underlying these apparent dissemination bottlenecks are unknown. Most *Yersinia* species contain *rhs* loci with associated orphan gene pairs, raising the possibility that clonal expansion through *rhs* recombination and growth selection may be a general feature of many enterobacterial infections. Rearrangement could function as a stochastic switch that enables some cells to deploy Rhs-CT^orphan^ and thereby “differentiate” into cells that are specialized for tissue invasion or immune modulation. Although Rhs-mediated inhibition clearly occurs between bacteria, it is also possible that Rhs toxins act directly as virulence factors. The C-terminal region of RhsT from *Pseudomonas aeruginosa* was recently shown to be delivered into mouse host cells [26]. In the process, the Rhs fragment activates the inflammasome and contributes to pathogenicity.

## Materials and Methods

### Strains and growth conditions

Bacterial strains were derived from *Salmonella enterica* serovar Typhimurium LT2 (*St*LT2) and are listed in Table S2. Bacteria were grown in LB medium [27] supplemented with 50 mM potassium phosphate (pH 7.3). Bacteria were incubated at 37°C with shaking at 200 rpm. Where appropriate, media were supplemented with antibiotics at the following concentrations: ampicillin (Amp), 200 mg/L; chloramphenicol (Cam), 17 mg/L; kanamycin (Kan), 80 mg/L; and tetracycline (Tet), 5 mg/L. Six independent lineages of *St*LT2 were obtained by serial passage for ∼1,000 generations [7]. Each lineage was passaged daily by dilution of 1.5 µL of overnight culture into 1.5 mL of fresh LB medium. Each evolved lineage was sampled periodically (100–150 generations) and stored at −80°C.

All growth competitions were conducted using ancestral *St*LT2 marked with the *flhC::cat* allele, which confers Cam resistance. Non-inhibitory clones isolated from the evolved cultures were transduced with the *flhC::cat* allele prior to testing for resistance. Ancestral and evolved cells were co-cultured in LB medium supplemented with 50 mM potassium phosphate (pH 7.3) at 37°C with shaking. At time 0 h, ∼10^6^ cfu (1 µL of overnight culture) from evolved and ancestral cultures were suspended in 2 mL of fresh LB (pH ∼7.3) and plated for viable cell counts before shaking incubation for 24 h at 37°C. After 24 h of co-culture, viable cell counts were determined by plating onto LB agar (to enumerate evolved and ancestral cells) and LB agar supplemented with Cam (to enumerate ancestral cells). The competitive index was calculated as the ratio of ancestral∶evolved cells at time 24 h divided by the cell ratio at 0 h. Ancestral *St*LT2 *flhC::cat* cells were also supplemented with either *rhsI^main^* or *rhsI^orphan^* on the chromosome under control of the *lac* promoter and on plasmid pBR322 under the *tet* promoter. Chromosomal *rhsI* ^orphan^ and plasmid-borne *rhsI^orphan^* individually provided partial protection against the evolved lineages (data not shown), but both copies were required for full immunity. Proximity-dependence of growth inhibition was determined as described previously [9]. Cells were grown to OD_600_ ∼0.3, then transferred to a *trans*-well culture plate (BD diagnostics) that separates the two populations with filter containing 0.4 µm (no-contact) or 8.0 µm (contact) pores. *Trans*-well culture plates were seeded at an evolved∶ancestral cell ratio of 1∶1 and incubated at 37°C with shaking for 24 h. Cultures were then plated onto selective media to determine viable cell counts and to calculate competitive indices.

### Construction of plasmids and chromosomal inserts

All oligonucleotides used in this study are presented in Table S3. The *rhsI^main^* and *rhsI^orphan^* genes were amplified from ancestral *St*LT2 chromosomal DNA using oligonucleotides 2337/2338 and 2340/2544 (respectively) and ligated to plasmid pBR322 using EcoRV and SalI restriction sites. The immunity genes were also placed under the *lac* promoter at the *glmS* locus using bacteriophage λ Red-mediated recombination [28]. Integration constructs containing *rhsI* genes flanked by a Kan-resistance cassette and *glmS*-derived homology regions were constructed by overlapping end-PCR as described previously [29]. The following primer pairs were used to amplify: upstream *glmS* homology (2666/2676), *lac* promoter (2677/2678 for *rhsI^main^* and 2677/2682 for *rhsI^orphan^*), *rhsI^main^* (2679/2680) or *rhsI^orphan^* (2683/2684), Kan-resistance cassette (2618/2619) and downstream *glmS* homology (2681/2667). The final PCR product was electroporated into *St*LT2 cells that express Red recombinase proteins, and transformants were selected on LB supplemented with Kan. Integrated immunity genes were verified by PCR analysis using primers 2666/2667 and subsequent DNA sequencing. The *flhC::cat* and STM0292::*kan* alleles were generated by PCR using primers 2436/2437 and 2410/2490 to amplify the *cat/kan* cassettes of plasmids pKD3 and pKD4, respectively. Each PCR product was integrated into the *St*LT2 chromosome by Red-mediated recombination.

To evaluate toxin activity and the specificity of immunity, individual *rhs-CT* and *rhsI* sequences were cloned under the control of inducible promoters on compatible plasmids. The *rhs-CT^main^* and *rhs-CT^orphan^* coding sequences were amplified with primers Sty-rhs(E1203)-Nco/Sty-rhs-Xho and Sty-rhs(E1203)-Nco/Sty-orph-rhs-Xho (respectively) and ligated to plasmid pCH450 [30] using NcoI and XhoI restriction sites. The *rhsI^main^* and *rhsI^orphan^* genes were amplified and ligated to a derivative of plasmid pTrc99A using KpnI and XhoI restriction sites. Rhs-CT^orphan^ was expressed and purified as a non-toxic variant fused to His_6_-tagged thioredoxin. The *his_6_-trxA* sequence was amplified from plasmid pSH21P::*trxA* [31] using primers pET-Sph and trxA-Bam-TEV-Kpn. The product was digested with SphI/BamHI and ligated to plasmid pET21b to generate plasmid pSH21P::*trxA-TEV*. The coding sequences for Rhs-CT^orphan^ residues 112–247 and RhsI^orphan^ were amplified using primers Sty-rhs(D1225)-Kpn/Sty-orph-rhsI-Xho) and the His208Ala mutation made by mega-primer PCR using oligonucleotide Sty-CTo1-H208A. The final product was digested with KpnI/XhoI and ligated to plasmid pSH21P::*trxA-TEV* to generate plasmid pCH10068. The resulting construct was used to overproduce His_6_-TrxA-Rhs-CT(H208A)^orphan^ fusion protein.

### Chromosomal DNA analysis

Chromosomal DNAs were isolated using the Sigma genomic DNA kit and digested with HincII restriction endonuclease. Digested DNAs were resolved by electrophoresis on 0.7% agarose gels at 34V for 15 h and blotted onto nylon membranes by capillary transfer. A probe to nucleotides 2969–3128 of *rhs^main^* was generated by PCR using oligos 2226/2227 and labeled with [^32^P]-labeled using the Prime-It Random Primer Labeling Kit (Agilent Technologies). Southern blots were visualized by phosphor imaging. Fragment sizes were calculated using a standard curve based on HindIII digested λ ladder (New England Biolabs, USA) run on the same gel. The proportion of *rhs* recombination junctions was determined by quantitative real-time PCR (qPCR) using oligonucleotides 2226/2231 using the cycle threshold C_t_-value method according to the manufacturer (Bio-Rad). Fluorescence was monitored on-line using the MyIQ iCycler real-time PCR system (Bio-Rad). The *rhs-rhs^orphan^* junction DNA levels were calculated relative to *bamA* DNA (oligos 1981/1990) in each sample and normalized to the level of junction DNA in ancestral cells.

### Antiserum preparation and immunoblot analysis

His_6_-TrxA-Rhs-CT(H208A)^orphan^ fusion protein was overproduced in *E. coli* CH2016 and purified by Ni^2+^-affinity chromatography as described [32]. The Rhs-CT(H208A)^orphan^ domain was released by TEV protease digestion and used for antiserum production in rabbits (CoCalico Biologicals). Non-specific antibodies were removed by incubation with carbonyl-diimidazole-activated agarose beads linked to soluble protein from *E. coli* strain CH2016 [33]. Briefly, protein-linked beads were resuspended in 0.5 mL of antiserum (1∶5 dilution) and mixed by rotation for 1 h at room temperature followed by additional incubation for 3 h at 4°C. This process was repeated at least four times with fresh beads.

Evolved lineages were grown to mid-log phase in LB medium supplemented with 50 mM potassium phosphate (pH 7.3) and cells were collected by centrifugation and frozen at −80°C. Cell pellets were resuspended in NuPage LDS-sample buffer (Invitrogen) at 70°C and treated with benzonase to degrade nucleic acids. Cell lysates were run on 3–7% NuPage Tris-acetate gradient gels (Novex) for the detection of the Rhs^main^-Rhs-CT^orphan^ chimera, or on 4–10% Precise Tris-glycine gradient gels (Thermo Scientific) to detect Rhs-CT^orphan^. Gels were electrotransferred to nitrocellulose membranes and the blots incubated with polyclonal antisera against Rhs-CT^orphan^ (1∶1,000 dilution) and secondary anti-rabbit 800CW antiserum (1∶10,000 dilution). Immunoblots were visualized using an Odyssey CLx Infrared Imaging System (LI-COR).

### Immunofluorescence microscopy and flow cytometry

Cells were incubated overnight with 4% formaldehyde in 0.15 M phosphate buffered saline (PBS, pH = 7.2). Cells were washed three times with PBS and incubated with polyclonal antibodies to Rhs-CT^orphan^ (1∶50 dilution) for 30 min. Cells were washed with PBS before incubation with secondary anti-rabbit Alexa-Fluor^480^ antibodies (1∶500 dilution) (Invitrogen) for 30 min on ice. After washing with PBS, cells were applied to poly-D-lysine coated slides, treated with Fluoro-gel II/DAPI (Electron Microscopy Sciences) and visualized by fluorescence microscopy. The fraction of evolved cells expressing Rhs-CT^orphan^ on the surface was determined by flow cytometry. Antibody-labeled cells were analyzed (50,000 events for each sample) with an Accuri C6 flow cytometer with gates set to include bacteria-sized particles. *St*LT2 Δ*rhs-CT^orphan^* cells were used to assess non-specific binding of the Rhs-CT^orphan^ antisera. The fraction of cells with surface Rhs-CT^orphan^ antigen was calculated as the ratio of green fluorescent particles in the population after subtracting background fluorescence observed with *St*LT2 Δ*rhs-CT^orphan^* cells.

## Supporting Information

Figure S1 Evolved *St*LT2 outcompete ancestral cells. The indicated evolved *St*LT2 lineages were co-cultured with the ancestral strain for 24 h in broth. Viable cell counts for each population were determined as colony forming units and these data were used to calculate the competitive index as described in Methods. **A**) Culture-evolved lineages after 1000-generations of growth in LB were competed against ancestral wild type cells. **B**) Mouse evolved lineages after 150-generations of growth in mice were competed against ancestral cells. Reported values represent the mean ± SEM for at least three independent experiments.
(PDF)

Figure S2 Growth rates of ancestral and evolved *St*LT2 strains. The growth rates of evolved lineages are expressed relative to the growth rate of ancestral cells, which was set to 1. Reported values represent the mean ± SEM for at least three independent experiments.
(PDF)

Figure S3 Variability of growth inhibitory activities of evolved inhibitor clones. Culture-evolved lineages were streaked on LB agar plates to obtain individual colonies. Ten colonies from each lineage were competed against the ancestral *St*LT2 strain as described for Figure 1. Reported values represent the mean ± SEM for at least two independent experiments. The hatched lines in each panel indicate an arbitrary cut-off (C.I. = 10^−1^) for whether a clone was considered to express growth inhibitory activity or not. Clones with C.I. error bars that cross the hatched line were considered to express growth inhibitory activity.
(PDF)

Figure S4 Evolved cells express Rhs^orphan-CT^ on the cell surface. Immunofluorescence analysis of ancestral cells and evolved lineages using antibodies against Rhs-CT^orphan^. Non-permeabilized cells were fluorescently labeled with antibodies to Rhs-CT^orphan^ as described in Methods. The Δ*rhs-CT^orphan^* cells carry a deletion of the *rhs-CT^orphan^* gene. P*ara*::Rhs-CT^orphan^ carry a plasmid encoded Rhs-CT^orphan^ under an arabinose inducible promoter. Scale is 10 µm×10 µm for each image. Cells were grown under inducing conditions as described in Methods.
(PDF)

Figure S5 Representative flow cytometry data for quantitation of the fraction of cells expressing cell surface Rhs^orphan-CT^. Non-permeabilized cells were fluorescently labeled using antibodies against Rhs-CT^orphan^ protein as described in Methods. The Δ*rhs-CT^orphan^* cells carry a deletion of the *rhs-CT^orphan^* gene. P*ara*::Rhs-CT^orphan^ carry a plasmid encoded Rhs-CT^orphan^ under an arabinose inducible promoter. Cells were grown under inducing conditions as described in Methods. Cells (50,000 events per sample) were analyzed using an Accuri C6 flow cytometer as described in Methods.
(PDF)

Figure S6 Rhs-CT sequence types from *Salmonella* isolates. **A**) 222 *Salmonella rhs* gene sequence from over 150 *Salmonella* isolates encode 57 different predicted Rhs-CT toxin sequences. Sequences are grouped together according to sequence homology, with Taylor coloring for amino acids. Sequence starts at the conserved DPxGL (boxed) demarking the beginning of the Rhs-CT. Orphan toxins are indicted by lowercase “o” in the sequence identifier. Numbers following the “o” indicate the position of the corresponding gene in the orphan cluster. **B**) Orphan Rhs-CT toxins are found on full-length Rhs proteins. The *St*LT2 *rhs-CT^orphan^* coding sequence is fused to *rhs^main^* in *Salmonella* serovar Saintpaul str. SARA23 as well as several serovar Newport strains (see panel A).
(PDF)

Table S1 *Salmonella enterica* strains used for *rhs-CT* analysis.
(XLSX)

Table S2 Strains and plasmids used in this study.
(DOCX)

Table S3 Oligonucleotides used in this study.
(DOCX)
