# Supplementary figures and images for: Selection of Orphan Rhs Toxin Expression in Evolved Salmonella enterica Serovar Typhimurium
Source: PLoS Genet. 2014 Mar 27;10(3):e1004255. doi: 10.1371/journal.pgen.1004255 (PMC3967940; doi:10.1371/journal.pgen.1004255)

Fig. S1

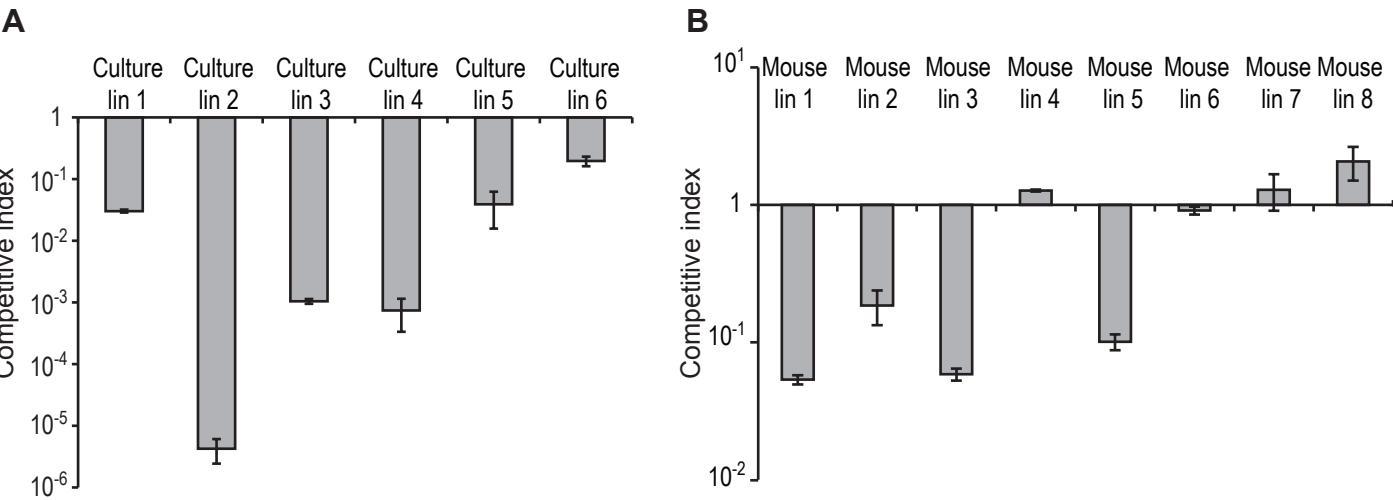

Supplement: Figure S1 — Evolved StLT2 outcompete ancestral cells. The indicated evolved StLT2 lineages were co-cultured with the ancestral strain for 24 h in broth. Viable cell counts for each population were determined as colony forming units and these data were used to calculate the competitive index as described in Methods. A) Culture-evolved lineages after 1000-generations of growth in LB were competed against ancestral wild type cells. B) Mouse evolved lineages after 150-generations of growth in mice were competed against ancestral cells. Reported values represent the mean ± SEM for at least three independent experiments. (PDF) [file pgen.1004255.s001.pdf]

**Fig. S2**

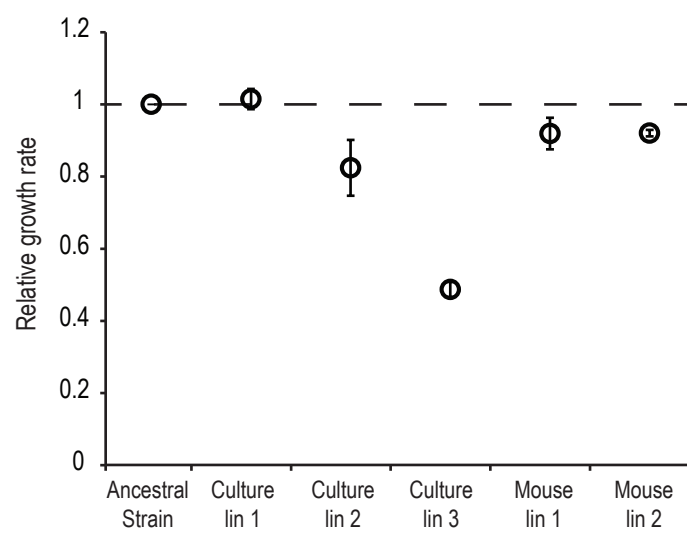

Supplement: Figure S2 — Growth rates of ancestral and evolved StLT2 strains. The growth rates of evolved lineages are expressed relative to the growth rate of ancestral cells, which was set to 1. Reported values represent the mean ± SEM for at least three independent experiments. (PDF) [file pgen.1004255.s002.pdf]

Fig. S3

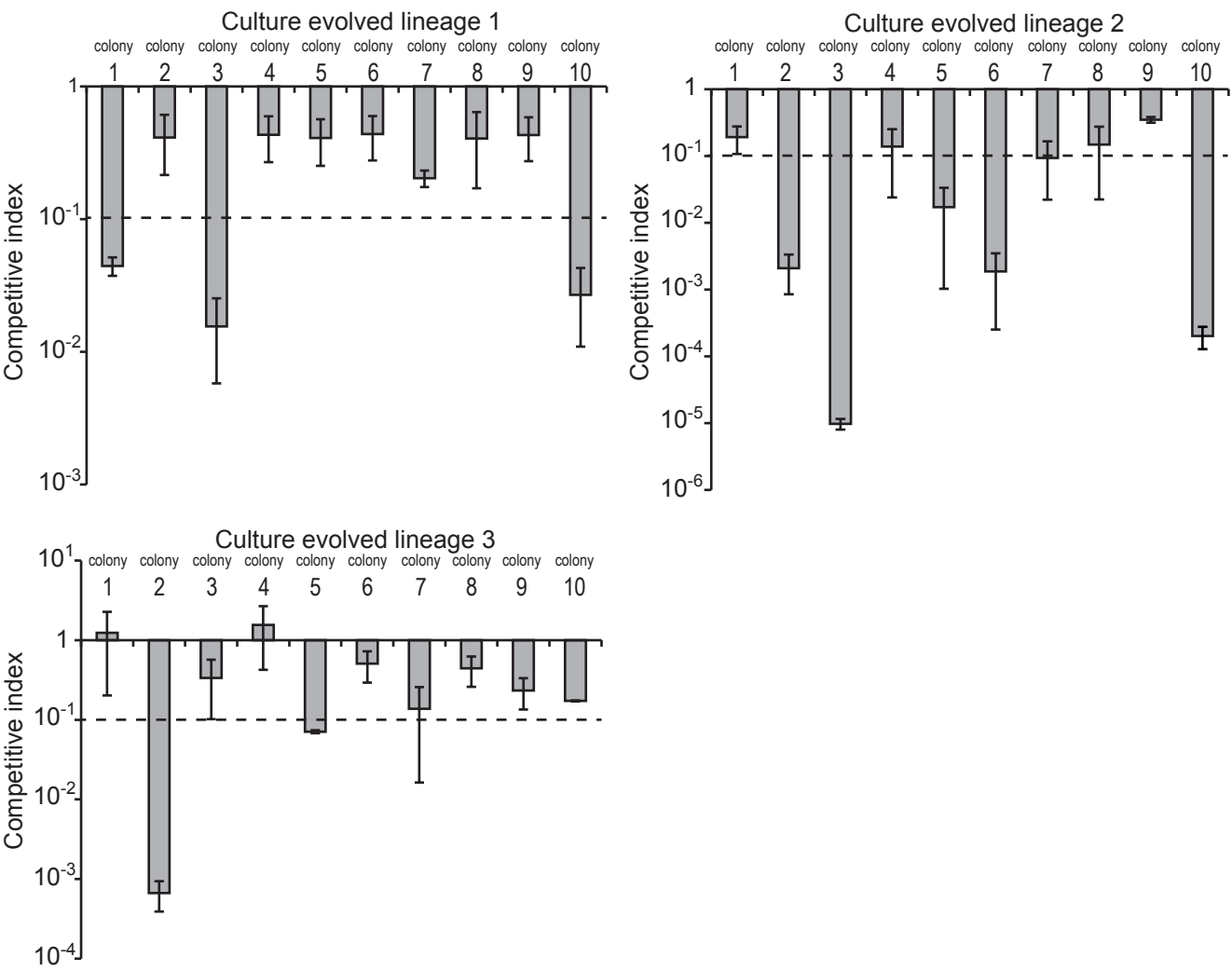

Supplement: Figure S3 — Variability of growth inhibitory activities of evolved inhibitor clones. Culture-evolved lineages were streaked on LB agar plates to obtain individual colonies. Ten colonies from each lineage were competed against the ancestral StLT2 strain as described for Figure 1. Reported values represent the mean ± SEM for at least two independent experiments. The hatched lines in each panel indicate an arbitrary cut-off (C.I. = 10−1) for whether a clone was considered to express growth inhibitory activity or not. Clones with C.I. error bars that cross the hatched line were considered to express growth inhibitory activity. (PDF) [file pgen.1004255.s003.pdf]

Fig. S4

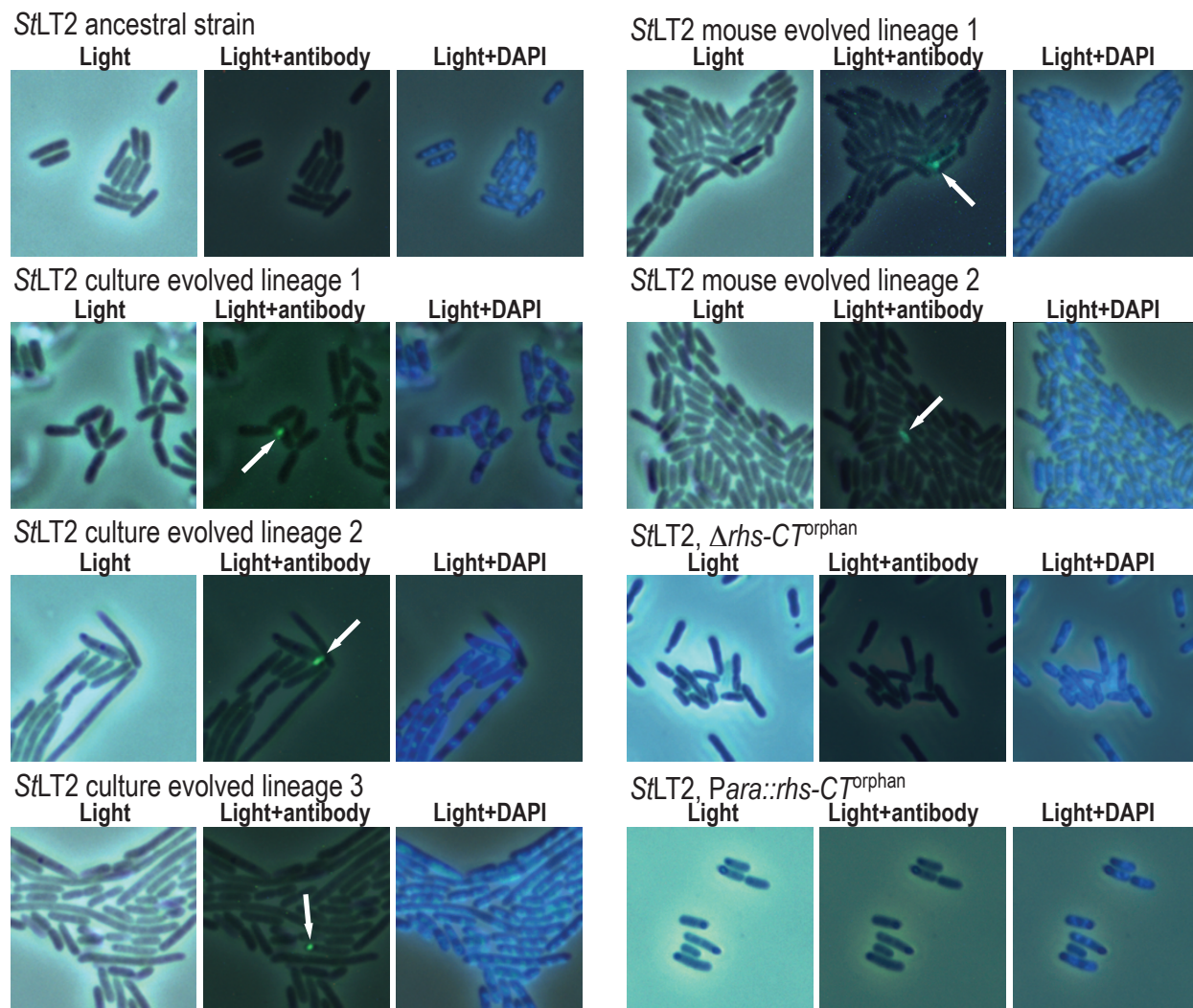

Supplement: Figure S4 — Evolved cells express Rhsorphan-CT on the cell surface. Immunofluorescence analysis of ancestral cells and evolved lineages using antibodies against Rhs-CTorphan. Non-permeabilized cells were fluorescently labeled with antibodies to Rhs-CTorphan as described in Methods. The Δrhs-CTorphan cells carry a deletion of the rhs-CTorphan gene. Para::Rhs-CTorphan carry a plasmid encoded Rhs-CTorphan under an arabinose inducible promoter. Scale is 10 µm×10 µm for each image. Cells were grown under inducing conditions as described in Methods. (PDF) [file pgen.1004255.s004.pdf]

Fig. S5

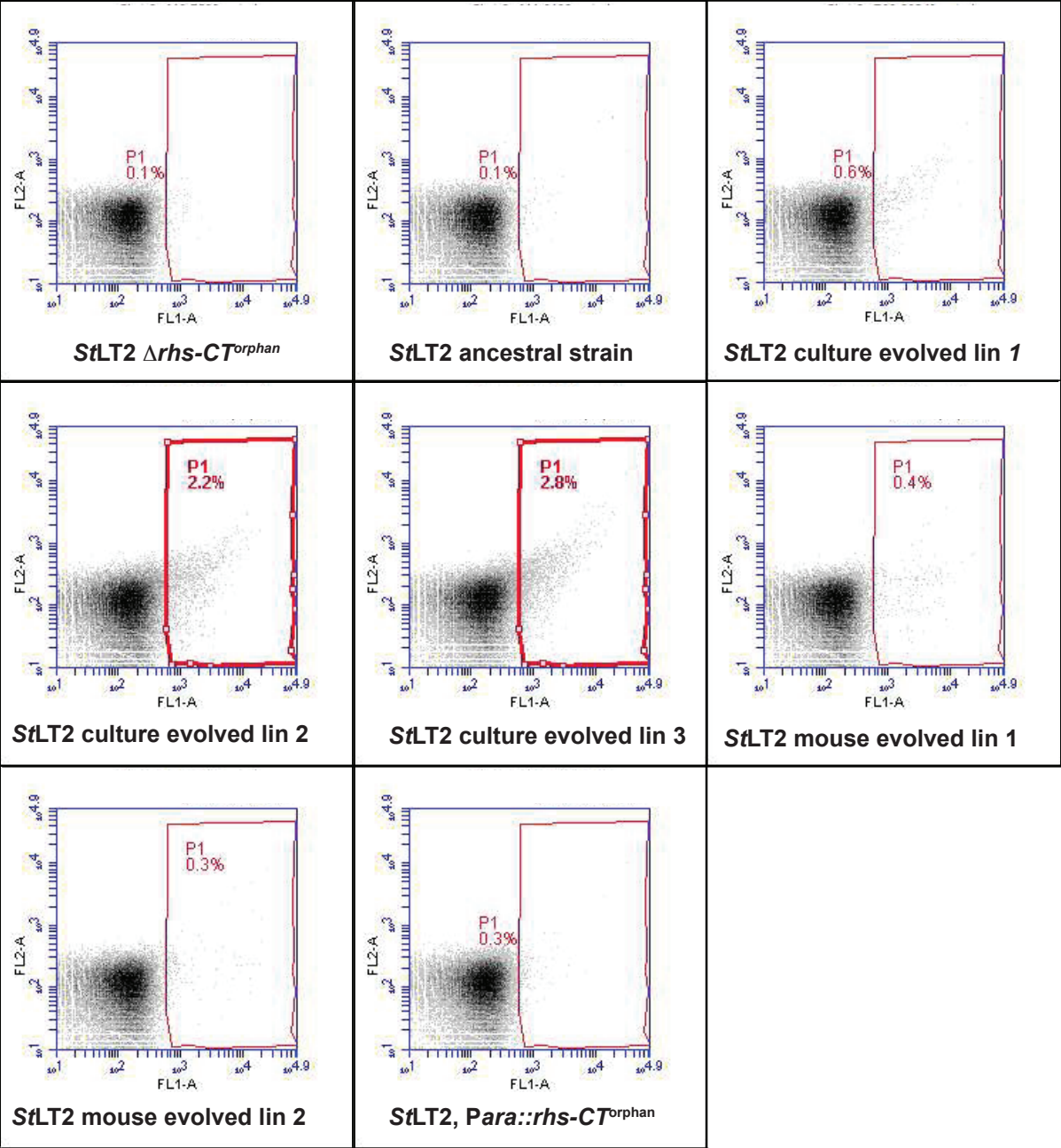

Supplement: Figure S5 — Representative flow cytometry data for quantitation of the fraction of cells expressing cell surface Rhsorphan-CT. Non-permeabilized cells were fluorescently labeled using antibodies against Rhs-CTorphan protein as described in Methods. The Δrhs-CTorphan cells carry a deletion of the rhs-CTorphan gene. Para::Rhs-CTorphan carry a plasmid encoded Rhs-CTorphan under an arabinose inducible promoter. Cells were grown under inducing conditions as described in Methods. Cells (50,000 events per sample) were analyzed using an Accuri C6 flow cytometer as described in Methods. (PDF) [file pgen.1004255.s005.pdf]

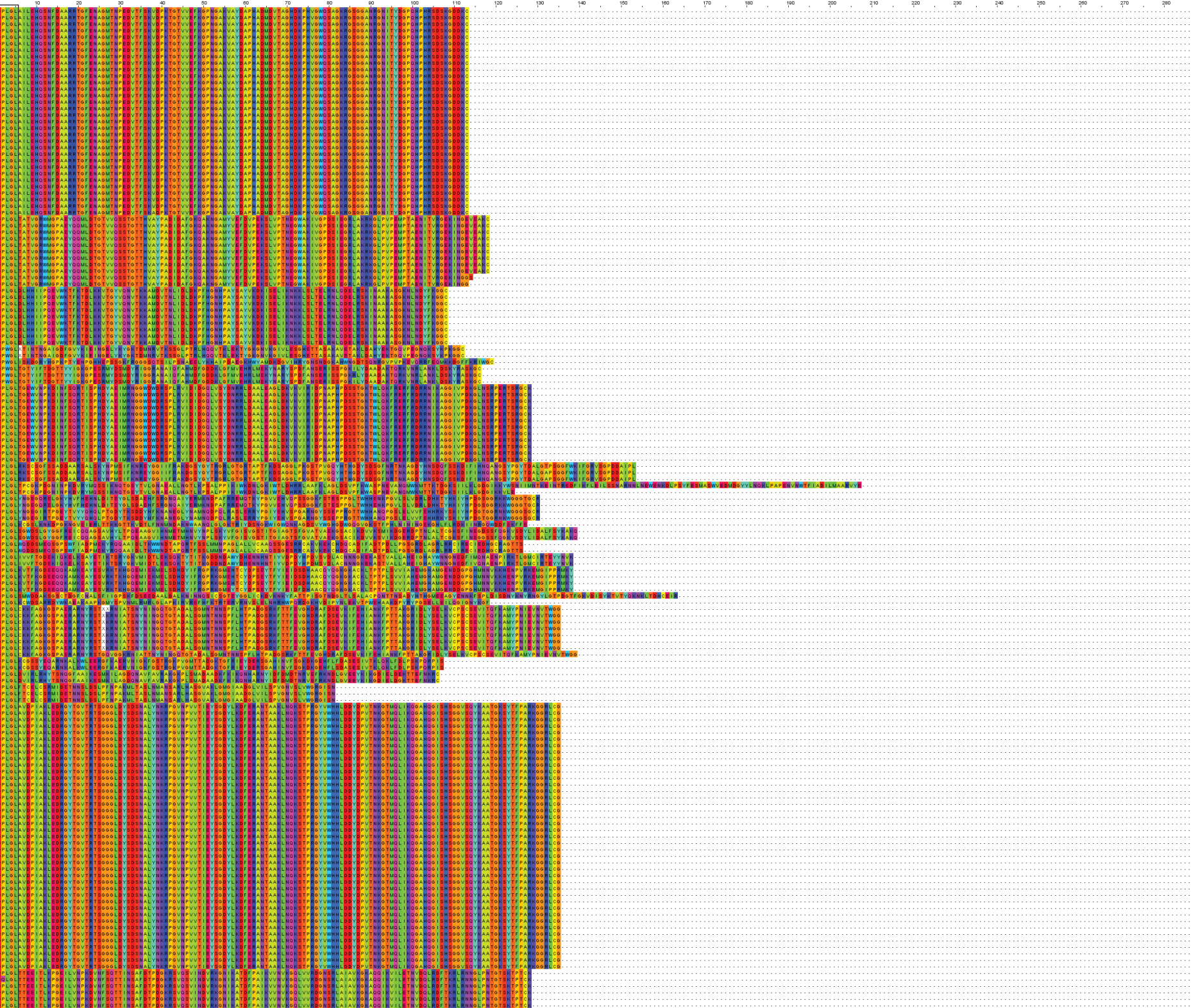

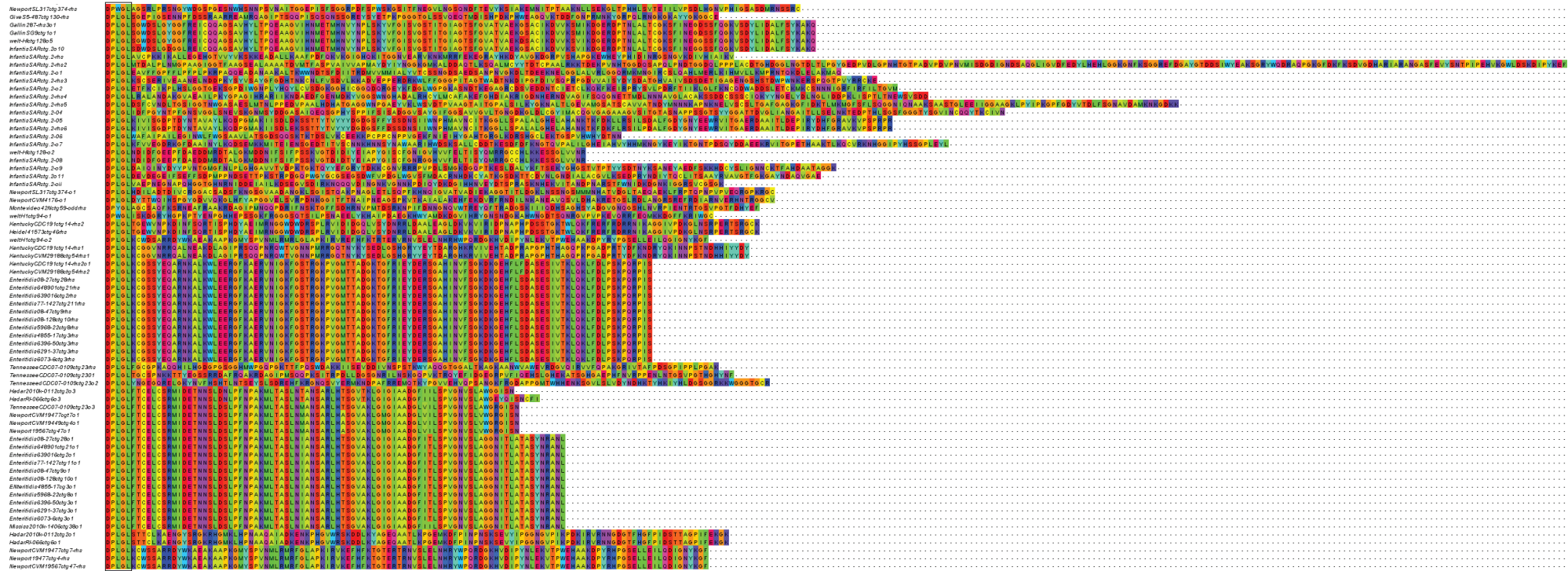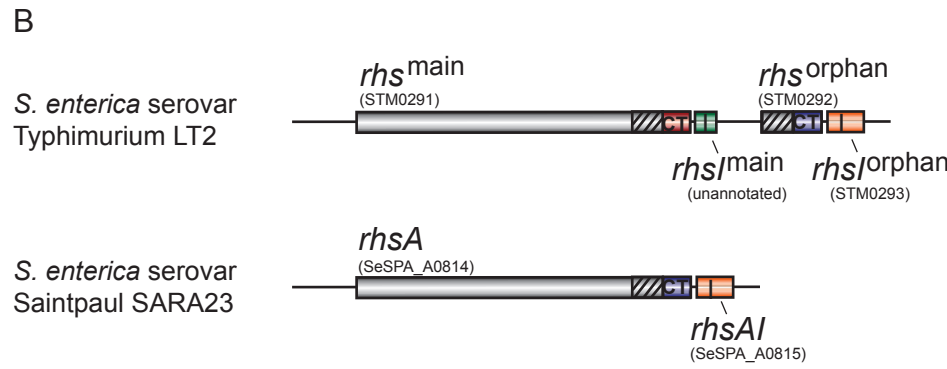

Supplement: Figure S6 — Rhs-CT sequence types from Salmonella isolates. A) 222 Salmonella rhs gene sequence from over 150 Salmonella isolates encode 57 different predicted Rhs-CT toxin sequences. Sequences are grouped together according to sequence homology, with Taylor coloring for amino acids. Sequence starts at the conserved DPxGL (boxed) demarking the beginning of the Rhs-CT. Orphan toxins are indicted by lowercase “o” in the sequence identifier. Numbers following the “o” indicate the position of the corresponding gene in the orphan cluster. B) Orphan Rhs-CT toxins are found on full-length Rhs proteins. The StLT2 rhs-CTorphan coding sequence is fused to rhsmain in Salmonella serovar Saintpaul str. SARA23 as well as several serovar Newport strains (see panel A). (PDF) [file pgen.1004255.s006.pdf]
