# Supplementary material for: Selection of Orphan Rhs Toxin Expression in Evolved Salmonella enterica Serovar Typhimurium
Source: PLoS Genet. 2014 Mar 27;10(3):e1004255. doi: 10.1371/journal.pgen.1004255 (PMC3967940; doi:10.1371/journal.pgen.1004255)
Supplement: Table S2 — Strains and plasmids used in this study. (DOCX) [file pgen.1004255.s008.docx]

**Table S2. Strains and plasmids used in this study.**

| **Strains** | **Description** | **Source or Reference** |
| --- | --- | --- |
| DA6192 | Wild type *Salmonella enterica* serovar Typhimurium LT2 | D. Andersson |
| DA20840 | Culture evolved DA6192 lineage 1 | ([1](#_ENREF_1)) |
| DA20841 | Culture evolved DA6192 lineage 2 | ([1](#_ENREF_1)) |
| DA20842 | Culture evolved DA6192 lineage 3 | ([1](#_ENREF_1)) |
| DA20843 | Culture evolved DA6192 lineage 4 | ([1](#_ENREF_1)) |
| DA20844 | Culture evolved DA6192 lineage 5 | ([1](#_ENREF_1)) |
| DA28045 | Culture evolved DA6192 lineage 6 | ([1](#_ENREF_1)) |
| DA5803 | Mouse evolved DA6192 lineage 1 | ([2](#_ENREF_2)) |
| DA5810 | Mouse evolved DA6192 lineage 2 | ([2](#_ENREF_2)) |
| DA5816 | Mouse evolved DA6192 lineage 3 | ([2](#_ENREF_2)) |
| DA5822 | Mouse evolved DA6192 lineage 4 | ([2](#_ENREF_2)) |
| DA5828 | Mouse evolved DA6192 lineage 5 | ([2](#_ENREF_2)) |
| DA5884 | Mouse evolved DA6192 lineage 6 | ([2](#_ENREF_2)) |
| DA5894 | Mouse evolved DA6192 lineage 7 | ([2](#_ENREF_2)) |
| DA5915 | Mouse evolved DA6192 lineage 8 | ([2](#_ENREF_2)) |
| DL6653 | DA6192 *flhC::cat* | This study |
| DL7133 | DA6192 *flhC*::*cat*, *glmS::kan-*P*_lac_*-*rhsI^main^*/pDAL923 | This study |
| DL7135 | DA6192 *flhC::cat, glmS::kan*-P*_lac_*-*rhsI^orphan^*/pDAL938 | This study |
| DL6756 | DA6192 *flhC*::Mu*d*J, STM0291-0292 fusion (*rhs^main^-rhs-CT^orphan^* fusion) | This study |
| DL7566 | DA6192 ∆*rhs-CT^orphan^::kan* | This study |
| CH2016 | X90 (DE3) ∆*rna ∆slyD::kan* | ([3](#_ENREF_3)) |
| **Plasmids** | **Description** | **Source or Reference** |
| pDAL923 | pBR322::*rhsI^main^*, main immunity gene cloned under the P*_tet_* promoter in plasmid pBR322 | This study |
| pDAL938 | pBR322::*rhsI^orphan^*, orphan immunity gene cloned under the P*_tet_* promoter in plasmid pBR322 | This study |
| pCH6329 | pSH21P::*trxA* | ([4](#_ENREF_4)) |
| pCH9229 | pTrc99*::rhsI^orphan^*, orphan immunity gene cloned under the IPTG-inducible P*_trc_* promoter | This study |
| pCH9230 | pTrc99*::rhsI^main^*, main immunity gene cloned under the IPTG-inducible P*_trc_* promoter | This study |
| pCH9277 | pCH450::*rhs-CT^main^*, main *rhs-CT* sequence cloned under the arabinose-inducible P_BAD_ promoter | This study |
| pCH9278 | pCH450::*rhs-CT^orphan^*, orphan *rhs-CT* gene cloned under the arabinose-inducible P_BAD_ promoter | This study |
| pCH10068 | pSH21P::*trxA-TEV-rhs-CT*(H208A)*^orphan^-rhsI^orphan^*, orphan *rhs-CT/rhsI* module fused to His_6_-tagged thioredoxin gene through TEV cleavage site | This study |
| Abbreviations: | | |

**References**

1. Koskiniemi S, Sun, S., Berg, O.G., Andersson, D.I. (2012) Selection-driven gene loss in bacteria. *PLoS genetics*.

2. Nilsson AI, Kugelberg E, Berg OG, & Andersson DI (2004) Experimental adaptation of Salmonella typhimurium to mice. *Genetics* 168(3):1119-1130.

3. Garza-Sanchez F, Gin JG, & Hayes CS (2008) Amino acid starvation and colicin D treatment induce A-site mRNA cleavage in Escherichia coli. *J Mol Biol* 378(3):505-519.

4. Ruhe ZC & Hayes CS (2010) The N-terminus of GalE induces tmRNA activity in Escherichia coli. *PLoS One* 5(12):e15207.
