# Supplementary material for: Selection of Orphan Rhs Toxin Expression in Evolved Salmonella enterica Serovar Typhimurium
Source: PLoS Genet. 2014 Mar 27;10(3):e1004255. doi: 10.1371/journal.pgen.1004255 (PMC3967940; doi:10.1371/journal.pgen.1004255)
Supplement: Table S3 — Oligonucleotides used in this study. (DOCX) [file pgen.1004255.s009.docx]

**Table S3. Oligonucleotides used in this study.**

| **Oligo** | **Sequence*^a^*** | **Reference** |
| --- | --- | --- |
| 2226 | 5´ - ACA GAA GCG GTT ACC TGA C | This study |
| 2227 | 5´ - CAC TCG TTA TAG CCG TAA C | This study |
| 2231 | 5´ - AGG GTT TGT CAT ACC CG CAT | This study |
| 2275 | 5´ - TAC CGA ACA TCA CGC CAA TC | This study |
| 2276 | 5´ - GTA TGA TGA GCC AGG CGA TG | This study |
| 2337 | 5´ - GCG CGA TAT CTG GGG AAG TTG AAG CAA AAT G | This study |
| 2338 | 5´ - ATA TGT CGA CAC TTC TTA CCC GGC CAA CTA | This study |
| 2340 | 5´ - ATA TGT CGA CCA CTC GCG CTT TAC TTA ATG G | This study |
| 2410 | 5´ - CGG AGT GTG GAC GGT TCG TCA GTC AGG ATC CGA TTG GGC TTG TAG GCT GGA GCT GCT TC | This study |
| 2413 | 5´ - TAT GAC AGG ATG CGC AGT CG | This study |
| 2436 | 5´ - TGA CTT ACC GCT GCT GGA GTG TTT GTC CAC ACC GTT TCG GCA TAT GAA TAT CCT CCT TA | This study |
| 2437 | 5´ - ATG AAG TGG ACG ATA CGG CGC GTA AGA AAA GGG CAT GAT ATG TAG GCT GGA GCT GCT TC | This study |
| 2490 | 5´ - GAC ATA TTT GAA TTT AAC ATT TAT CAT CTC CCT TAG AGT Cca tat gaa tat cct cct ta | This study |
| 2544 | 5´ - GCG CGA TAT CTA ACT GAC TCT AAG GGA GAT GAT AA | This study |
| 2618 | 5´ - TGT AGG CTG GAG CTG CTT C | This study |
| 2619 | 5´ - CAT ATG AAT ATC CTC CTT A | This study |
| 2666 | 5´ - ATT CCC GAA GAG TGG AAC GT | This study |
| 2667 | 5´ - AGG TCT GGA TGC GTC CAT TG | This study |
| 2676 | 5´ - CAG CTG CAT TAA TGA ATC GGG TAC AAA TGT AGT ACC AGG C | This study |
| 2677 | 5´ - CCG ATT CAT TAA TGC AGC TG | This study |
| 2678 | 5´ - TTT ATT TAG CAT TTC CTC CTT TCC TGT GTG AAA TTG TTA T | This study |
| 2679 | 5´ - AGG AGG AAA TGC TAA ATA AAT TTA AAT T | This study |
| 2680 | 5´ - GAA GCA GCT CCA GCC TAC ATT ATT TAA TAC TTA TCA TAA | This study |
| 2681 | 5´ - TAA GGA GGA TAT TCA TAT GTT ACT CTA CGG TAA CCG ATT | This study |
| 2682 | 5´ - TGA ATT TAA CAT TTC CTC CTT TCC TGT GTG AAA TTG TTA T | This study |
| 2683 | 5´ - AGG AGG AAA TGT TAA ATT CAA ATA TGT C | This study |
| 2684 | 5´ - GAA GCA GCT CCA GCC TAC ATT ACT TAA TGG TTG AGC ATA CA | This study |
| Sty-rhs (E1203)-Nco | 5´ - TTG CCA TGG AGA CAG GGC TGC ATT ACA ATC TG | This study |
| Sty-rhs-Xho | 5´ - ACC CTC GAG TTA AAT TTA TTT AGC | This study |
| Sty-orph-CT-Xho | 5´ - ATA CTC GAG TTT AAC ATT TAT CAT CTC | This study |
| Sty-rhsI-Kpn | 5´ - TGA GGT ACC ATG CTA AAT AAA TTT AAA TTG | This study |
| Sty-rhsI-Xho | 5´ - GGC CTC GAG TTA TTT AAT ACT TAT CAT AAA ATC | This study |
| Sty-orph-rhsI-Kpn | 5´ - AGA GGT ACC ATG TTA AAT TCA AAT ATG TC | This study |
| Sty-orph-rhsI-Xho | 5´ - GCA CTC GAG CTT TAC TTA ATG GTT GAG C | This study |
| Sty-rhs (D1225)-Kpn | 5´ - GTC GGT ACC GAT CCG ATC GGG CTG | This study |
| Sty-CTo1-H208A | 5´ - GGG CAT AAA CCA GCT GTT GGT TGG CAA TCC G | This study |
| pET-Sph | 5´ - CAA GGA ATG GTG CAT GCC TGC AGA TGG CGC CC | This study |
| trxA-Bam-TEV-Kpn | 5´ - AAA GGT ACC AGA CTG AAA ATA CAG GTT CTC AGA AGT GGA TCC CGC CAG GTT AGC GTC G | This study |
| *^a^*Restriction endonuclease sites are underlined. | | |
